# MCP1 Inverts the Correlation between FGF23 and Omega 6/3 Ratio: Is It Also True in Renal Transplantation?

**DOI:** 10.3390/jcm12185928

**Published:** 2023-09-12

**Authors:** Deborah Mattinzoli, Stefano Turolo, Masami Ikehata, Simone Vettoretti, Giovanni Montini, Carlo Agostoni, Costanza Conti, Matteo Benedetti, Piergiorgio Messa, Carlo Maria Alfieri, Giuseppe Castellano

**Affiliations:** 1 Renal Research Laboratory, Fondazione IRCCS Ca’ Granda Ospedale Maggiore Policlinico, 20122 Milan, Italy; 2 Pediatric Nephrology, Dialysis and Transplant Unit, Fondazione IRCCS Ca’ Granda Ospedale Maggiore Policlinico, 20122 Milan, Italy; 3 Department of Nephrology, Dialysis and Renal Transplantation, Fondazione IRCCS Ca’ Granda Ospedale Maggiore Policlinico, 20122 Milan, Italy; 4 Department of Clinical Sciences and Community Health, University of Milan, 20122 Milan, Italy; 5 Pediatric-Immunorheumatology Unit, Fondazione IRCCS Ca’ Granda Ospedale Maggiore Policlinico, 20122 Milan, Italy; 6 Post-Graduate School of Specialization in Nephrology, University of Milan, 20157 Milan, Italy

**Keywords:** fibroblast growth factor 23, kidney transplant, polyunsaturated fatty acids, monocyte chemoattractant protein 1

## Abstract

During chronic kidney disease (CKD) progression, an increase in fibroblast growth factor (FGF23) is present. In stage 5, a positive correlation between FGF23 and omega-6 (n-6) polyunsaturated fatty acids (PUFAs) emerges. Hypothesizing that the rising positive correlation between monocyte chemoattractant protein 1 (MCP1) and n-6 in stage 4 could be the cause, we previously explored FGF23 and MCP1’s roles in dyslipidemia and cardiovascular risk in CKD. In the present paper, we retraced the study evaluating 40 kidney transplant patients (KTx), a cohort where several factors might modify the previous relationships found. An ELISA and gas chromatography assessed the MCP1, FGF23, and PUFA levels. Despite the FGF23 increase (*p* < 0.0001), low MCP1 levels were found. A decrease in the n-6/n-3 ratio (*p* = 0.042 CKD stage 4 vs. 5) lowered by the increase in both n-3 αlinolenic (*p* = 0.012) and docosapentaenoic acid (*p* = 0.049) was observed. A negative correlation between FGF23 and the n-6/n-3 ratio in CKD stage 4 (r^2^ −0.3 *p* = 0.043) and none with MCP1 appeared. According to our findings, different mechanisms in the relationship between FGF23, PUFAs, and MCP1 in CKD and KTx patients might be present, which is possibly related to the immunosuppressive status of the last. Future research will further clarify our hypothesis.

## 1. Introduction

End-stage renal disease has doubled in the last decade, and the benefits of kidney transplantation (KTx) over dialysis are undisputed in terms of improved survival, quality of life, and cost [1,2].

KTx is associated with a significant reduction in cardiovascular (CV) risk compared to dialysis [3,4]. However, KTx patients remain at a higher CV risk than the general population. In addition to the evaluation and correction of the so-called “traditional CV risk factors”, attention is focused on the assessment of “non-traditional CV factors”, such as inflammation and oxidative stress, as well as KTx-specific CV risk factors [5,6,7].

In this context, our group, following the increasingly convincing concept that pro-inflammatory factors may play a key role in the genesis of CV events in CKD, described an association between fibroblast growth factor 23 (FGF23) and the pro-inflammatory factor monocyte chemoattractant protein 1 (MCP1) in causing dyslipidemia [8].

The first factor, FGF23, which is increased early in CKD to compensate for the mineral imbalance, contributes directly to CV outcomes through cardiac, vascular, and inflammatory disease [8,9,10,11,12,13,14,15]. FGF23 is a 251 amino acid protein phosphorylated to the S180 amino acid by FAM20C kinase, which induces its proteolysis by the FURIN convertase, producing its c-terminal inactivated fragment. In contrast, the lack of phosphorylation of FGF23 makes it susceptible to O-glycosylation at T178 by GALNT3, resulting in the only intact and biologically active form being produced [16]. Under normal conditions, the whole length and the fragment are produced; an imbalance occurs in some pathological diseases, such as chronic kidney disease [17]. David’s group reports that inflammation affects the complex network of regulatory factors of FGF23 [18]. In particular, the group describes that while the induction of acute inflammation by injecting bacteria or interleukin 6 (IL6) in mice increases the circulating level of only cFGF23 without an effect on iFGF23, in chronic inflammation, they increase both. Considering the intricate mechanisms connected to the inflammation and regulation of FGF23, we decided to analyze both iFGF and its cleaved form.

The second potent chemotactic factor, monocyte chemoattractant protein 1 (MCP1), perfectly complements the first and is stimulated by inflammatory cytokines, growth factors, and oxidative stress conditions [19,20].

The well-recognized critical direct or indirect (receptor-mediated) role in the development of CV disease (atherosclerotic plaque formation, ischemia/reperfusion injury, transplant rejection) identifies MCP1 as a potential therapeutic target [21,22,23,24].

Regarding dyslipidemia, several articles describe the importance of the balance between the omega-6 (n-6) precursors of some potent inflammatory mediators of CKD and their recognized counterpart, omega-3 (n-3) polyunsaturated fatty acids (PUFAs), suggesting the useful n-6/n-3 ratio as a biomarker of CV and chronic disease [25,26,27].

As chronic inflammation in CKD plays a crucial role in strong dyslipidemic CV risk, we previously observed an increase in FGF23, MCP1, and n-6/n-3 during eGFR decline. We also found an inversion of the correlation between FGF23 and n-6/n-3 fatty acids, specifically positive in stage 3 and negative in stage 5. We then hypothesized that this inversion was triggered by the increase in MCP1 in intermediate CKD stage 4, which is positively correlated with both FGF23 and total omega-6 (n-6) polyunsaturated fatty acid (PUFA) [8].

To validate our hypothesis, in the present pilot study, we repeated the same experiment in patients with CKD in patients with KTx, which is considered an excellent clinical condition, to investigate the risk factor of the reciprocal relationship between reduced renal function and CV risk; indeed, immunosuppressive therapy causes the onset of hypertension in more than 50% of patients, mainly due to dyslipidemia [28,29,30].

Replicating our previous study in patients with KTx in whom immunosuppressive therapy could counteract the possible triggering of MCP1 could validate our hypothesis regarding its possible role in modifying the FGF23-n-6/n-3 correlation.

## 2. Materials and Methods

### 2.1. Patients

All enrolled 40 kidney transplanted (KTx) patients > 18 years old were recruited between 2019 and 2020 at our institution’s (Fondazione IRCCS Ca’ Granda Ospedale Maggiore Policlinico) Department of Nephrology, Dialysis, and Renal Transplantation. To obtain 40 patients for our pilot study, no sample size analyses were performed, and the recruitment was ended after the inclusion of the 40th patient. In agreement with the clinical protocols of our department, immunosuppressive therapy was mainly composed of (a) steroids: n = 34, prednisone mean dosage 6.25 ± 3.0 mg/die; (b) calcineurin inhibitors: cyclosporine n = 8 mean dosage 118 ± 35 mg/die—tacrolimus n = 29 mean dosage 5 ± 3 mg/die; and mycophenolate: mycophenolate mofetil n = 25 mean dosage 1272 ± 428 mg/die—sodium mycophenolate n = 10 mean dosage 720 ± 169 mg/die. Inhibitors of mTOR were prescribed in 4 patients: sirolimus n = 3 mean dosage 1.6 ± 0.2 mg/die—everolimus n = 1 at a daily dosage of 1.25 mg.

Participants’ blood samples were collected on the morning of the same day of the visit after an overnight fast of at least eight hours for clinical and laboratory evaluations. In addition, 24 h urine samples were collected for routine analysis. Glomerular filtration rate (eGFR) was estimated through the CKD-EPI formula.

According to eGFR values, KTx patients were considered affected by CKD stage 3: 60 mL/min > GFR > 30 mL/min; CKD stage 4: 30 mL/min > GFR > 15 mL/min; CKD stage 5: GFR < 15 mL/min. According to eGFR values, KTx patients were affected by CKD stage 3 if their eGFR was between 60 and 30 mL/min/1.73 m^2^. Patients with an eGFR between 30 and 15 mL/min/1.73 m^2^ were considered affected by CKD stage 4; CKD stage 5 was attributed to patients with eGFR under 15 mL/min/1.73 m^2^. Exclusion criteria were KTx patients affected by active cancer, symptomatic infectious diseases in the previous two months, decompensated chronic liver disease, heart failure (NYHA II-IV), endocrine diseases disorders other than diabetes mellitus and mineral metabolism abnormalities, hospitalization in the last two months, inability to cooperate. All patients with an assumed overall life expectancy of <6 months were also excluded. Ethical approval: This study was conducted following ICP’s good clinical practice guidelines, the Declaration of Helsinki, and the approval of our institution’s ethics committee (approval document 347/2010, PROVE: Proteinuria and Vascular Endpoints). All patients signed informed consent to participate in this study as specified in the ICMJE recommendations.

### 2.2. Fatty Acid Analysis

Serum aliquots of 50 µL were collected during a routine control. For fatty acids methylation, 800 µL of 3N hydrochloric acid solution (Sigma-Aldrich, St. Louis, MO, USA) was added in methanol; then, the samples were incubated for 1 h at 90 °C. The samples were refrigerated at 4 °C for 10 min. Next, 2 mL of semi-saturated potassium chloride solution (KCl) and 350 µL of hexane (Sigma-Aldrich) were added. The samples were vortexed and then centrifuged at 3000× *g* rpm for 10 min at 15 °C. Finally, the hexane layer (the top layer) was collected from each vial and transferred to a gas chromatography vial for fatty acid (FA) profile evaluation with a Shimadzu Nexis GC-2030 gas chromatograph (Shimadzu, Kyoto, Japan) equipped with a 30 m FAMEWAX Restek fused silica capillary column (Restek, Bellefonte, PA, USA). Gas chromatography results were analyzed with Lab Solution 5.97 SP1 software (Shimadzu, Japan). Both individuals and groups of FAs (PUFA, PUFA n-3, and PUFA n-6) are expressed as relative percentages of the total FAs considered.

During a routine control visit, serum aliquots of 50 µL were collected. The fatty acids (FAs) analysis was performed, starting with the sample methylation. A total of 50 µL of serum was put into Pyrex vials with 800 µL of 3N hydrochloric acid solution (Sigma-Aldrich, St. Louis, MO, USA) in methanol. Then, the samples were incubated for 1 h at 90 °C. After incubation, samples were refrigerated at 4 °C for 10 min. In the next step, 2 mL of semi-saturated potassium chloride solution (KCl) and 350 µL of hexane (Sigma-Aldrich) were added to each sample. The vials were vortexed for at least 20 s and then centrifuged at 3000× *g* rpm for 10 min at 15 °C.

Finally, the hexane layer (the top layer) was collected from each vial and transferred to a gas chromatography vial for fatty acid profile evaluation with a Shimadzu Nexis GC-2030 gas chromatograph (Shimadzu, Kyoto, Japan) equipped with a 30 m FAMEWAX Restek fused silica capillary column (Restek, Bellefonte, PA, USA). The resulting chromatograms were analyzed with Lab Solution 5.97 SP1 software (Shimadzu, Japan). Both individuals and groups of FAs (PUFA, PUFA n-3, and PUFA n-6) are expressed as relative percentages of the total FAs considered.

### 2.3. Enzyme-Linked Immunosorbent Assay (ELISA) for Monocyte Chemoattractant Protein 1 (MCP1) and FGF23 Intact/c-Terminal

Blood samples were centrifuged for 10 min at 3500× *g* rpm to separate serum and plasma and stored immediately at −80 °C until assay. Serum levels of MCP1 were measured with a commercially available ELISA kit (R&D Systems, Minneapolis, MN, USA) according to the manufacturer’s instructions.

The mean minimum detectable dose was 1.7 pg/mL, and the coefficients of variation for intra-assay and inter-assay on serum samples were 4.7–7.8% and 4.6–6.7%, respectively. Plasma concentrations of intact/terminal FGF23 were also measured using the commercially available kit (Immutopics, Inc., San Clemente, CA, USA). The lowest detectable concentration of intact FGF23 was 1.5 pg/mL, and the coefficients of variation for intra-assay and inter-assay were 2.0–4.1% and 3.5–9.1%, respectively. The lowest measurable human FGF23 c-terminal concentration was 1.5 RU/mL, and the coefficients of variation for intra-assay and inter-assay were 1.4–2.4% and 2.4–4.7%, respectively.

### 2.4. Statistical Analysis

All descriptive tables report data as mean and standard deviation unless otherwise stated. *t*-test analysis assessed differences between demographic and clinical biomarkers; Kruskal–Walli’s test assessed differences between CKD groups; graphs also show the total median line for each parameter considered. Data were correlated by two-tailed Spearman’s bivariate analysis. Negative correlations are expressed with the prefix “(−).” The bivariate correlation plot also shows a 95% regression. A *p* < 0.05 was considered statistically significant for all analyses. All analyses were performed with SPSS v.21 software (IBM, Armonk, NY, USA).

## 3. Results

The cohort studied was characterized by 40 patients. The transplant vintage of the cohort examined was a median of 1043 days [min 13–max 11,291 days]. Concerning pre-transplant nephropathy, 11 patients were affected by glomerular diseases, 8 had a genetic disease, and 4 had diabetic nephropathy. Finally, in three and four cases, a urologic disease or other types of nephropathies were respectively present. In eight cases, the basal nephropathy was not identified. In two patients, a previous history of cardiovascular disease (atrial fibrillation and venous thrombosis) was present.

Table 1 shows the demographic, clinical, and key biomarker data of the analyzed cohort of 40 patients. The patients were divided according to their CKD stage: 23 in stage 3, 13 in stage 4, and 4 in stage 5.

Table 1 shows the overall cohort’s demographic, clinical, and key biomarker data according to their CKD stage: 23 in stage 3, 13 in stage 4, and 4 in stage 5.

The mean age of the overall cohort was 55 ± 16 years, and 55% of the KTx patients were male. The mean value of the eGFR was 34 ± 16 mL/min. Regarding the renal function parameters, the eGFR decreased, and plasma creatinine increased significantly from CKD 3 to 5 (for both *p* < 0.001). In line with the KTx status, the mean phosphorus levels were generally low, whereas the lipid parameters were mainly in range. The comparison between stage 3 and stage 5 values showed a significant reduction in Ca levels (*p* = 0.009), and no significant difference was observed for Na and K.

Compared to the CKD population studied in our previous research, the levels of MCP1 in CKD were 465 ± 159.59 pg/mL vs. the present value in Ktx patients: 241 ± 104 pg/mL (*p* > 0.0001).

As for the biomarkers analyzed, both i/cFGF23 increased (*p* < 0.0001, *p =* 0.018 respectively) from CKD stage 3 to 5, whereas no significant differences were found for MCP1 levels (Table 1).

The PUFA profile among the CKD stages was then analyzed and reported in Table 2. No increase in total PUFAs was observed among the CKD stages, including the AA/LA ratio, an inflammation parameter associated with CVD. When n-3 PUFA was examined in detail, no significant trend towards an increase in total n-3 was observed. However, a significant increase in n-3 alpha-linolenic acid (ALA) was observed from CKD 3 to 5 (*p* = 0.012) and in docosapentaenoic acid (DPA) from CKD 3 to 5 (*p* = 0.049) and CKD 4 to 5 (*p* = 0.019). Regarding n-6, no significant trend towards a decrease was observed in total and in each n-6 PUFA analyzed (Table 2).

**Figure 1:**
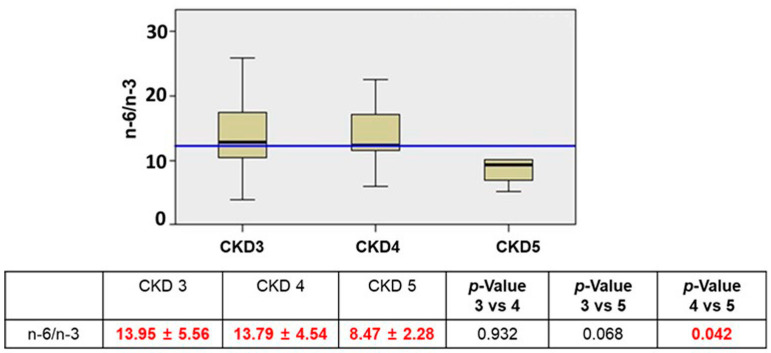
The n-6/n-3 trend data according to CKD stage. Blue line = median (n = 23 in CKD3, n = 13 in CKD4, n = 4 in CKD5). The values in red indicate that this marker decrease during the CKD stage.

Therefore, analyzing the n-6/n-3 ratio, which is considered a helpful benchmark for the analysis of PUFA profile balance, compared to our previous study in the CKD population in which a significant increase occurred (*p* = 0.03 from the median), a significant decrease in KTx patients was observed from CKD stage 4 to 5 (*p* = 0.042) following the previous increase of n-3 observed in the levels (Figure 1).

We analyzed each correlation to investigate further the relationship between i/cFGF23, PUFA, and MCP1.

**Figure 2:**
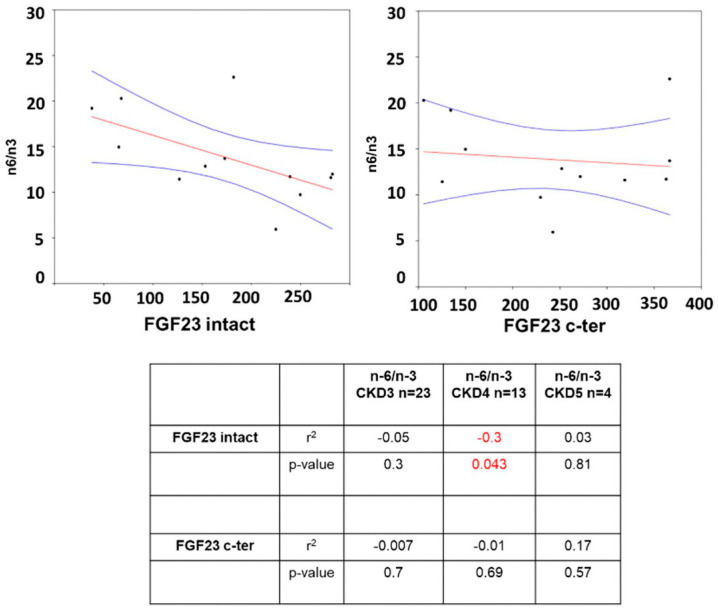
Graphs representing correlation between n-6/n-3 and i/cFGF23 respectively among CKD stage 4. Table representing the correlation between n-6/n-3 and i/cFGF23 among CKD stages by Two-tailed Spearman bivariate analysis. Significant negative correlation in red indicated by “−”.

Starting with analyzing a possible correlation between PUFA and FGF23, only negative correlations appeared in CKD stage 4 with iFGF23 (r^2^ −0.3 *p =* 0.043) (Figure 2). Furthermore, there was no correlation between the AA/LA ratio and iFGF23 (CKD3 r^2^ 0.006 *p* = 0.7, CKD4 r^2^ 0.0001 *p* = 0.96, and CKD5 r^2^ 0.84 *p* = 0.08) or with cFGF23 (CKD3 r^2^ −0.03 *p* = 0.38, CKD4 r^2^ −0.06 *p* = 0.43, and CKD5 r^2^ 0.48 *p* = 0.3) between CKD stages.

Therefore, there was no significant correlation between PUFA and MCP1 during renal decline (Table 3). There was also no correlation between MCP1 and the AA/LA ratio.

**Figure 3:**
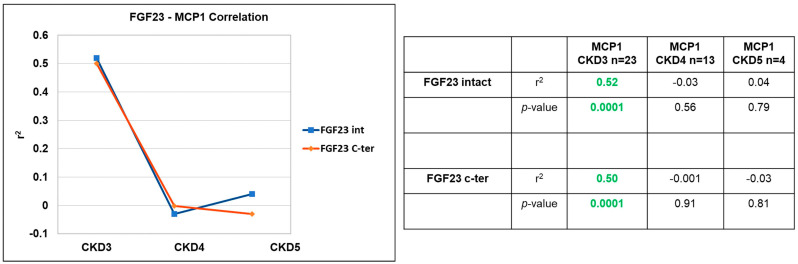
Correlation between i/cFGF23 and MCP1 during CKD stages 3, 4, and 5. Two-tailed Spearman bivariate analysis. n = 23 in CKD3, n = 13 in CKD4, n = 5 in CKD5. Significant positive correlation in green.

Finally, the analysis of i/cFGF23 and MCP1 was performed. A direct correlation was found only in CKD stage 3 with both i/cFGF23s (r^2^ 0.5 *p* = 0.0001 for both) (Figure 3).

## 4. Discussion

The significant advantages of transplantation over dialysis are well-known. However, it is well-known that the reason lies in improving the CV system [31]. CV disease accelerates the progression of CKD to end-stage, and intervention of non-traditional factors, such as inflammation, has been shown to prevent and partially reduce the damage of traditional factors, such as dyslipidemia [32].

In this context, our recent study focusing on CKD patients described an association between the biomarker FGF23, the n-6/n-3 PUFA ratio, and the chemokine MCP1 [8]. Specifically, we reported a negative correlation between FGF23 and the n-6/n-3 ratio in CKD stage 3, which became utterly positive in CKD stage 5. We therefore hypothesized that MCP1 and its emerging positive correlation with all n-6 PUFAs in the intermediate “switch stage” 4 CKD may trigger this detrimental inverse correlation [8].

To evaluate the repeatability of our findings in different renal disease conditions, we then planned to repeat the same steps of the study in a cohort of kidney transplant recipients, hypothesizing an influence of immunosuppressive therapy on patients’ MCP1 levels.

As a first step in our study, we evaluated the immunosuppressant’s effect on MCP1 levels. Despite the increase in FGF23 i/c, no change in MCP1 was observed, which was in contrast to the increase observed in our previous study in CKD patients.

The role of FGF23 in enhancing inflammatory activation in hepatocytes via both PLCγ/calcineurin/NFAT signaling and nuclear translocation of NFκB is well-established, as its directly and indirectly involved in enhancing MCP1 levels [12,33,34]. Immunosuppressive agents such as calcineurin inhibitors or tacrolimus can interfere with the nuclear translocation of NFAT and NFKB and significantly reduce monocyte production of chemokines and interleukins, including MCP1 [35,36,37,38].

The relationship between MCP1, FGF23, and inflammation hides further intricate mechanisms. MCP1 attracts circulating monocytes to inflamed tissue, and the monocyte-derived macrophage called M0 could be polarized by several external stimuli in pro-inflammatory M1 cells or anti-inflammatory M2 ones [39]. The exhibition of the M1 phenotype by several factors (e.g., interferon-gamma, tumor necrosis factor α (TNFα), lipopolysaccharide) leads to anti-bacterial, anti-virus, and anti-tumoral activity and the induction of type II diabetes, atherosclerosis, and autoimmune disease. Conversely, the M2 feature of several factors (e.g., IL4, 10, 12), despite the immunosuppression, tissue repair, and angiogenesis properties, could induce tumor progression, fibrosis, and allergies [40,41,42]. In this panorama, FGF23, produced by M1 macrophages via the NFκB and JAK/STAT1 pathways, acts as a paracrine factor stimulating TNFα in M0 and then promoting M1 polarization and inhibiting the arginase-1 expression responsible for the activation in the M2 macrophages leading to the 1,25(OH)2 D production. An FGF23 counterregulatory action occurred in M2 through 1,25(OH)2 D, blocking the arginase-1 suppression and the TNFα production responsible for FGF23 production in M1 [43]. Moreover, MCP1 itself could play a paracrine role in this mechanism. Indeed, its abrogation reduces NFκB and TNFα action, increasing M2 polarization [44]. Furthermore, while arachidonic acid inhibits M2 macrophage polarization, its metabolites stimulate it [45].

Overall, the intricate role of FGF23, MCP1, and FA in macrophage polarization regulating pro-inflammatory and anti-inflammatory macrophage functions during CKD progression should not be underestimated, enhancing the reason for our previous and present study offering interesting yet unclear questions that can and should be elucidated in future studies.

In the presence of a steady increase in FGF23, maintaining a low level of MCP1 would have allowed us to verify its hypothetical role and involvement not only in increasing pro-inflammatory n-6 PUFAs but also in establishing the positive correlation between FGF23 and the n-6/n-3 PUFA ratio described in our previous study [8].

We then focused on the PUFA profile, and despite the increase in FGF23, no change in n-6 PUFAs occurred. Conversely, a partial non-significant increase in total “CV protective” n-3 despite the significant increase was detected only in n-3 ALA and DPA individually in the KTx patients.

Only a partial unsurprisingly increase in n-3 was observed when the PUFA synthesis pathway was considered. Indeed, EPA derives from ALA and then is metabolized to DPA. A reduction in the EPA level could be the result of three different events: it can be due to a decrease in EPA synthesis from ALA (and a consequent increase in ALA levels); it can be due to an increased DPA level (and a consequent resulting increase rise in DPA levels); or it could be the result of increased EPA metabolization, which could not affect the ALA or DPA level but could increase the concentration of EPA metabolites.

Then, considering the complicated event in FA metabolism, the helpful n-6/n-3 parameter has usually been viewed, where the increase in the n-6/n-3 ratio is associated with the atherosclerotic process triggered by a pro-inflammatory environment. In contrast, a reduction of the ratio is desirable for modulating the inflammatory event in a positive way, possibly reducing CV risk [46].

Looking at the level of the n-6/n-3 PUFA ratio, a significant decrease was found during the renal decline in patients with KTx in contrast to our previous study on CKD.

Then, the starting increase of n-3 seems to avoid the detrimental effect of increasing the n-6 series, which is mainly due to the conversion of arachidonic acid (AA) into some potent eicosanoid inflammatory mediators of CKD (e.g., prostaglandins, thromboxane, and leukotrienes) contributing to the progression of CKD [47,48].

To evaluate the hypothesized activation of the cascade towards AA production, the activity of the FADS enzymes responsible for the conversion of LA into AA is often considered. This is assessed by calculating the parameter AA/LA ratio, which indicates a pro-inflammatory environment highly prone to the onset of pathological cardiovascular processes [49]. In the present study, the AA/LA ratio analysis does not show any increase, confirming our data.

In our previous article on patients with CKD in whom FGF23 increased steadily as in the KTx patients, the landscape was completely reversed with a significant decrease in CV-protective n-3 from CKD 3 to 4 and a significant increase in pro-inflammatory n-6 from CKD 3 to 5 and from CKD 4 to 5.

From this contrasting result obtained in the two different CKD populations analyzed, we assumed that the role of FGF23 in increasing n-6 and decreasing n-3 was attenuated when MCP1 was kept low by the immunosuppressive therapy in KTx.

Analyzing the correlation between MCP1 and PUFAs, in CKD patients, we observed an increasing direct correlation of MCP1 with some n-6s (DGLA, Osbond, ARA, GLA) and a negative correlation with n-3 between stages, while in KTx patients, no correlation was observed following the fact that the absence of increased MCP1 could avoid its influence on the PUFA balance in favor of the pro-inflammatory one. The lack of correlation between AA/LA and MCP1 supports our data.

Regarding the correlation between i/cFGF23 and MCP1, if a positive correlation occurred previously in CKD patients, now in KTx patients, we observed that only a positive correlation in CKD stage 3 disappears in CKD stage 5. We hypothesized that in CKD stage 3 with relatively low FGF23 levels, keeping MCP1 low with immunosuppressive therapy makes the correlation positive, which is lost when FGF23 increases and MCP1 is kept stable.

Finally, we analyzed the correlation between FGF23 and PUFA. While in CKD patients, we obtained an inverse positive correlation between FGF23 and n-6/n-3, in KTx patients, we observed a negative correlation between FGF23 and the n-6/n-3 ratio in CKD stage 4. Again, there was no correlation between FGF23 and AA/LA.

The answer to why only stage 4 shows a negative correlation may lie in the n6/n3 ratio difference observed across the CKD stages. Indeed, while no change in n-6/n-3 occurred from stages 3 to 4, a significative reduction was observed from CKD stages 4 to 5. Then, we hypothesized that the renal functional status at stage 4 causes a strong increase in FGF23, whose action on MCP1 (and may not be the only one), which is responsible for n-6 growth, was reduced by the immunosuppressive therapy. Therefore, the n-6 pro-inflammatory pathway block caused their reduction and the negative correlation in CKD stage 4 between FGF23 and n-6/n-3. In CKD stage 5, the negative correlation was probably maintained: Future studies including more patients might confirm this hypothesis.

This last lack in CKD stage 5 is irrelevant, however, considering that the blockage of the mechanism occurring in stage 4 already answered the present work’s purpose.

Then, comparing the data obtained from the CKD patients treated and untreated with immunosuppressants, it appears that the increase in n-6/n-3 PUFAs exerted by FGF23 is indirect and could be mediated with MCP1. Eliminating MCP1 via immunosuppressive therapy avoids the pro-inflammatory effect of FGF23 that caused the increase in the n-6/n-3 ratio and the emergence of its positive correlation. The small cohort studied could be a limitation of this study, but the present research would only confirm our previous data; in any case, the strict inclusion and exclusion criteria guarantee a good homogeneity of the patients. Despite the limited number of patients studied, we decided not to group patients for different CKD stages. The different CKD stages depict different clinical conditions potentially characterized by varying degrees of additional, more impactful comorbidities (mineral metabolism abnormalities, anemia, inflammation). Strengths of this study are the criteria of patient selection that allowed us to obtain homogeneous subgroups and the comparison with our previous study that permitted a complete “vision” of the role of MCP1, FGF23, and their interaction with FA. The limitation of this study is the cohort size of the CKD5 subgroup, but being a pilot study regarding transplanted patients, this was considered during the study design step.

In conclusion, this pilot study, which replicates the previous one using the “human transplantation model” to better understand the mechanism related to dyslipidemia and inflammation, highlights the role of MCP1 as a trigger. Given the broad spectrum of actions underlying immunosuppressive therapy, MCP1 can be considered only one of the potential triggers for this mechanism. Given the present result, an anti-MCP1 strategy or a better understanding of the mechanism of immunotherapeutic agents may have potential therapeutic value to reduce the detrimental interrelationship between FGF23, dyslipidemia, and inflammation based on CV pathophysiological mechanisms in CKD. The reported possibility that MCP1 and its receptor may help to monitor kidney transplantation reinforces the importance of previous studies on MCP1 as a future predictor [50,51]. In a forthcoming paper, it would also be worthwhile to anticipate this study in the opposite direction by analyzing how immunosuppressive treatment alters the endogenous metabolism of FA, which is associated with or determines pro-inflammatory metabolites.

## Figures and Tables

**Table 1:** Demographic, clinical, and biomarker data among CKD stages 3, 4, 5.

	Total n = 40	CKD 3 n = 23	CKD 4 n = 13	CKD 5n = 4	*p*-Value 3 vs. 4	*p*-Value 3 vs. 5	*p*-Value 4 vs. 5
Demographic Data							
Age (years)	55 ± 16	53 ± 17	57 ± 17	62 ± 9	0.46	0.30	0.60
Gender m/f (N)	22/18	18/5	3/10	1/3			
Clinical Data							
eGFR (mL/min/1.73 m^2^)	34.44 ± 16.54	45.52 ± 13.66	23.48 ± 4.20	11.98 ± 3.06	<0.0001	<0.0001	<0.0001
P-Creat (mg/dL)	2.24 ± 0.96	1.73 ± 0.41	2.44 ± 0.44	4.33 ± 1.30	<0.0001	<0.0001	<0.0001
COL tot (mg/dL)	192.91 ± 30.12	187.26 ± 28.04	194.31 ± 33.28	215.25 ± 23.77	0.522	0.078	0.265
TGL (mg/dL)	164.77 ± 64.75	156.68 ± 51.65	163.54 ± 78.03	207.25 ± 75.71	0.766	0.114	0.340
HDL (mg/dL)	55.30 ± 13.42	55.21 ± 15.84	57.46 ± 9.58	48.75 ± 12.31	0.651	0.454	0.155
LDL (mg/dL)	101.55 ± 30.47	100.09 ± 22.17	96.47 ± 39.62	125.05 ± 27.29	0.743	0.062	0.202
Na (mmol/L)	141.63 ± 2.69	141.95 ± 1.78	141.62 ± 2.99	140.25 ± 1.50	0.696	0.091	0.400
K (mmol/L)	4.44 ± 0.46	4.48 ± 0.39	4.39 ± 0.49	4.40 ± 0.75	0.562	0.744	0.981
Biomarker Data							
Ca (mg/dL)	9.46 ± 0.72	9.63 ± 0.69	9.50 ± 0.57	8.52 ± 0.79	0.563	0.009	0.015
S-phosphorus (mg/dL)	3.43 ± 0.87	3.15 ± 0.59	3.62 ± 0.73	4.23 ± 1.80	0.055	0.036	0.320
FGF23 INT (pg/mL)	152.8 ± 113.90	108.0 ± 60.05	169.4 ± 82.97	345.0 ± 214.25	0.017	<0.0001	0.023
FGF23 CT (RU/mL)	242.6 ± 191.80	200.5 ± 191.70	239.3 ± 94.82	485.3 ± 284.57	0.502	0.018	0.013
MCP1 (pg/mL)	241.4 ± 104.10	247.7 ± 122.40	242.4 ± 77.56	203.2 ± 78.89	0.889	0.494	0.393

*p*-values regarding gender were calculated with a chi-square test; *p*-values regarding all other parameters were calculated with a *t*-test. Abbreviations: eGFR = estimated glomerular filtration rate, P-Creat = plasma creatinine, COL = cholesterol, TGL = triglyceride, HDL/LDL = high- and low-density lipoprotein, Na = sodium, K = potassium, Ca = calcium, S-phosphorus = serum phosphorus, FGF23 INT/CT = fibroblast growth factor 23 intact/C-terminal, MCP1 = monocyte chemoattractant protein 1. Data are expressed as mean ± standard deviation.

**Table 2:** Fatty acid profiles among CKD stages.

	CKD 3	CKD 4	CKD 5	*p*-Value 3 vs. 4	*p*-Value 3 vs. 5	*p*-Value 4 vs. 5
PUFA	35.37 ± 4.12	34.95 ± 3.82	33.80 ± 3.56	0.769	0.486	0.603
PUFA n-3	2.76 ± 1.37	2.61 ± 1.04	3.73 ± 1.01	0.739	0.192	0.076
ALA (18:3 n-3)	0.27 ± 0.10	0.35 ± 0.16	0.41 ± 0.06	0.101	**0.012**	0.435
EPA (20:5 n-3)	0.09 ± 0.14	0.06 ± 0.03	0.03 ± 0.01	0.607	0.411	0.060
DPA (22:5 n-3)	0.32 ± 0.17	0.31 ± 0.12	0.52 ± 0.19	0.796	**0.049**	**0.019**
DHA (22:6 n-3)	2.15 ± 1.22	1.94 ± 0.9	2.79 ± 0.81	0.596	0.333	0.116
PUFA n-6	32.53 ± 4.18	32.27 ± 3.41	30.00 ± 3.71	0.849	0.272	0.272
LA (18:2 n-6)	24.47 ± 4.65	24.10 ± 3.96	23.08 ± 1.87	0.814	0.568	0.631
GLA (18:3 n-6)	0.08 ± 0.07	0.09 ± 0.07	0.05 ± 0.03	0.776	0.411	0.319
DGLA (20:3 n-6)	1.44± 0.56	1.19 ± 0.39	1.08 ± 0.53	0.176	0.257	0.669
AA (20:4 n-6)	6.62 ± 2.03	6.97 ± 2.1	5.83 ± 2.43	0.632	0.495	0.373
AdA (22:4 n-6)	0.29 ± 0.12	0.28 ± 0.14	0.21 ± 0.10	0.853	0.251	0.388
Osbond (22:5 n-6)	0.08 ± 0.06	0.07 ± 0.07	0.05 ± 0.03	0.688	0.367	0.572
AA/LA	0.28 ± 0.11	0.33 ± 0.15	0.25 ± 0.11	0.240	0.700	0.370

The PUFA is expressed as a relative % of the total considered FA. Abbreviations: PUFA: polyunsaturated fatty acids, ALA: α linolenic acid, EPA: eicosapentaenoic acid, DPA: docosapentaenoic acid, DHA: docosahexaenoic acid, LA: linoleic acid, GLA: gamma linolenic, DGLA: dihomo-γ-linolenic acid, AA: arachidonic acid, AdA: adrenic acid. The values in green indicate that these markers increase during the CKD stage. The *p*-value was calculated with a *t*-test. n = 23 in CKD3, n = 13 in CKD4, n = 4 in CKD5).

**Table 3:** Correlation between PUFA and MCP1 among CKD stages.

Fatty Acid	MCP1 CKD3 n = 23		MCP1 CKD 4 n = 13		MCP1 CKD5 n = 4	
	r^2^	*p*-Value	r^2^	*p*-Value	r^2^	*p*-Value
PUFA	−0.330	0.144	0.070	0.829	<0.001	1.000
PUFA n-3	0.087	0.708	0.130	0.688	0.400	0.600
ALA (18:3 n-3)	−0.081	0.726	−0.040	0.901	−0.400	0.600
EPA (20:5 n-3)	0.030	0.400	−0.090	0.760	−0.010	0.890
DPA (22:5 n-3)	0.113	0.626	−0.344	0.274	0.800	0.200
DHA (22:6 n-3)	0.118	0.610	0.144	0.656	0.400	0.600
PUFA n-6	−0.425	0.055	−0.098	0.762	−0.400	0.600
LA (18:2 n-6)	−0.403	0.070	−0.154	0.632	−0.200	0.800
GLA (18:3 n-6)	<−0.001	0.910	−0.090	0.340	−0.770	0.120
DGLA (20:3 n-6)	−0.179	0.437	−0.259	0.416	<0.001	1.000
AA (20:4 n-6)	0.066	0.775	0.242	0.449	−0.400	0.600
AdA (22:4 n-6)	−0.030	0.380	−0.010	0.700	−0.040	0.790
Osbond (22:5 n-6)	0.140	0.078	−0.070	0.390	−0.530	0.260
n-6/n-3	−0.153	0.507	−0.123	0.704	−0.400	0.600
AA/LA	0.170	0.060	<0.001	0.990	0.010	0.880

*p* < 0.05 and “−” indicates a negative correlation. Correlation analysis by two-tailed Spearman bivariate analysis. n = 23 in CKD3, n = 13 in CKD4, n = 4 in CKD5.

## Data Availability

All data generated or analyzed during this study are included in this published article.

## References

[1] Laupacis A., Keown P., Pus N., Krueger H., Ferguson B., Wong C., Muirhead N. (1996). A Study of the Quality of Life and Cost-Utility of Renal Transplantation. Kidney Int..

[2] Thongprayoon C., Hansrivijit P., Leeaphorn N., Acharya P., Torres-Ortiz A., Kaewput W., Kovvuru K., Kanduri S.R., Bathini T., Cheungpasitporn W. (2020). Recent Advances and Clinical Outcomes of Kidney Transplantation. J. Clin. Med..

[3] Tonelli M., Wiebe N., Knoll G., Bello A., Browne S., Jadhav D., Klarenbach S., Gill J. (2011). Systematic Review: Kidney Transplantation Compared with Dialysis in Clinically Relevant Outcomes. Am. J. Transplant..

[4] Meier-Kriesche H.U., Schold J.D., Srinivas T.R., Reed A., Kaplan B. (2004). Kidney Transplantation Halts Cardiovascular Disease Progression in Patients with End-Stage Renal Disease. Am. J. Transplant..

[5] Jankowski J., Floege J., Fliser D., Böhm M., Marx N. (2021). Cardiovascular Disease in Chronic Kidney Disease: Pathophysiological Insights and Therapeutic Options. Circulation.

[6] Szlagor M., Dybiec J., Młynarska E., Rysz J., Franczyk B. (2023). Chronic Kidney Disease as a Comorbidity in Heart Failure. Int. J. Mol. Sci..

[7] Liu M., Li X.C., Lu L., Cao Y., Sun R.R., Chen S., Zhang P.Y. (2014). Cardiovascular Disease and Its Relationship with Chronic Kidney Disease. Eur. Rev. Med. Pharmacol. Sci..

[8] Mattinzoli D., Turolo S., Alfieri C.M., Ikehata M., Caldiroli L., Armelloni S., Montini G., Agostoni C., Messa P., Vettoretti S. (2022). MCP1 Could Mediate FGF23 and Omega 6/Omega 3 Correlation Inversion in CKD. J. Clin. Med..

[9] Rodríguez M., López I., Muñoz J., Aguilera-Tejero E., Almaden Y. (2012). FGF23 and Mineral Metabolism, Implications in CKD-MBD. Nefrologia.

[10] Batra J., Buttar R.S., Kaur P., Kreimerman J., Melamed M.L. (2016). FGF-23 and Cardiovascular Disease: Review of Literature. Curr. Opin. Endocrinol. Diabetes Obes..

[11] Vázquez-Sánchez S., Poveda J., Navarro-García J.A., González-Lafuente L., Rodríguez-Sánchez E., Ruilope L.M., Ruiz-Hurtado G. (2021). An Overview of FGF-23 as a Novel Candidate Biomarker of Cardiovascular Risk. Front. Physiol..

[12] Mattinzoli D., Li M., Castellano G., Ikehata M., Armelloni S., Elli F.M., Molinari P., Tsugawa K., Alfieri C.M., Messa P. (2022). Fibroblast Growth Factor 23 Level Modulates the Hepatocyte’s Alpha-2-HS-Glycoprotein Transcription through the Inflammatory Pathway TNFα/NFκB. Front. Med..

[13] Faul C., Amaral A.P., Oskouei B., Hu M.C., Sloan A., Isakova T., Gutiérrez O.M., Aguillon-Prada R., Lincoln J., Hare J.M. (2011). FGF23 Induces Left Ventricular Hypertrophy. J. Clin. Investig..

[14] Silswal N., Touchberry C.D., Daniel D.R., McCarthy D.L., Zhang S., Andresen J., Stubbs J.R., Wacker M.J. (2014). FGF23 Directly Impairs Endothelium-Dependent Vasorelaxation by Increasing Superoxide Levels and Reducing Nitric Oxide Bioavailability. Am. J. Physiol. Endocrinol. Metab..

[15] Mattinzoli D., Rastaldi M.P., Ikehata M., Armelloni S., Pignatari C., Giardino L.A., Li M., Alfieri C.M., Regalia A., Riccardi D. (2018). FGF23-Regulated Production of Fetuin-A (AHSG) in Osteocytes. BONE.

[16] Tagliabracci V.S., Engel J.L., Wiley S.E., Xiao J., Gonzalez D.J., Appaiah H.N., Koller A., Nizet V., White K.E., Dixon J.E. (2014). Dynamic Regulation of FGF23 by Fam20C Phosphorylation, GalNAc-T3 Glycosylation, and Furin Proteolysis. Proc. Natl. Acad. Sci. USA.

[17] Wolf M., White K.E. (2014). Coupling Fibroblast Growth Factor 23 Production and Cleavage: Iron Deficiency, Rickets, and Kidney Disease. Curr. Opin. Nephrol. Hypertens..

[18] David V., Martin A., Isakova T., Spaulding C., Qi L., Ramirez V., Zumbrennen-Bullough K.B., Sun C.C., Lin H.Y., Babitt J.L. (2016). Inflammation and Functional Iron Deficiency Regulate Fibroblast Growth Factor 23 Production. Kidney Int..

[19] Niu J., Kolattukudy P.E. (2009). Role of MCP-1 in Cardiovascular Disease: Molecular Mechanisms and Clinical Implications. Clin. Sci..

[20] Vianna H.R., Soares C.M.B.M., Silveira K.D., Elmiro G.S., Mendes P.M., De Sousa Tavares M., Teixeira M.M., Miranda D.M., Simões E Silva A.C. (2013). Cytokines in Chronic Kidney Disease: Potential Link of MCP-1 and Dyslipidemia in Glomerular Diseases. Pediatr. Nephrol..

[21] Piemonti L., Calori G., Lattuada G., Mercalli A., Ragogna F., Garancini M.P., Ruotolo G., Luzi L., Perseghin G. (2009). Association between Plasma Monocyte Chemoattractant Protein-1 Concentration and Cardiovascular Disease Mortality in Middle-Aged Diabetic and Nondiabetic Individuals. Diabetes Care.

[22] Stepanova N., Driianska V., Savchenko S. (2020). Dyslipidemia and Intraperitoneal Inflammation Axis in Peritoneal Dialysis Patients: A Cross-Sectional Pilot Study. Kidney Dis..

[23] Gosling J., Slaymaker S., Gu L., Tseng S., Zlot C.H., Young S.G., Rollins B.J., Charo I.F. (1999). MCP-1 Deficiency Reduces Susceptibility to Atherosclerosis in Mice That Overexpress Human Apolipoprotein B. J. Clin. Investig..

[24] Deshmane S.L., Kremlev S., Amini S., Sawaya B.E. (2009). Monocyte Chemoattractant Protein-1 (MCP-1): An Overview. J. Interferon Cytokine Res..

[25] Noels H., Lehrke M., Vanholder R., Jankowski J. (2021). Lipoproteins and Fatty Acids in Chronic Kidney Disease: Molecular and Metabolic Alterations. Nat. Rev. Nephrol..

[26] Simopoulos A.P. (2008). The Importance of the Omega-6/Omega-3 Fatty Acid Ratio in Cardiovascular Disease and Other Chronic Diseases. Exp. Biol. Med..

[27] Zhang Y., Sun Y., Yu Q., Song S., Brenna J.T., Shen Y., Ye K. (2023). Higher Ratio of Plasma Omega-6/Omega-3 Fatty Acids Is Associated with Greater Risk of All-Cause, Cancer, and Cardiovascular Mortality: A Population-Based Cohort Study in UK Biobank. medRxiv.

[28] Nowalany-Kozielska E., Wojnicz R. (2003). Immunosuppressive Therapy for Heart Failure: A Guide to Patient Selection. Am. J. Cardiovasc. Drugs.

[29] Miller L.W. (2002). Cardiovascular Toxicities of Immunosuppressive Agents. Am. J. Transplant..

[30] Kalnins A., Thomas M.N., Andrassy M., Müller S., Wagner A., Pratschke S., Rentsch M., Klussmann S., Kauke T., Angele M.K. (2015). Spiegelmer Inhibition of MCP-1/CCR2—Potential as an Adjunct Immunosuppressive Therapy in Transplantation. Scand. J. Immunol..

[31] Rangaswami J., Mathew R.O., Parasuraman R., Tantisattamo E., Lubetzky M., Rao S., Yaqub M.S., Birdwell K.A., Bennett W., Dalal P. (2019). Cardiovascular Disease in the Kidney Transplant Recipient: Epidemiology, Diagnosis and Management Strategies. Nephrol. Dial. Transplant..

[32] Menon V., Gul A., Sarnak M.J. (2005). Cardiovascular Risk Factors in Chronic Kidney Disease. Kidney Int..

[33] Rovin B.H., Dickerson J.A., Tan L.C., Hebert C.A. (1995). Activation of Nuclear Factor-Kappa B Correlates with MCP-1 Expression by Human Mesangial Cells. Kidney Int..

[34] Singh S., Grabner A., Yanucil C., Schramm K., Czaya B., Krick S., Czaja M.J., Bartz R., Abraham R., Di Marco G.S. (2016). Fibroblast Growth Factor 23 Directly Targets Hepatocytes to Promote Inflammation in Chronic Kidney Disease. Kidney Int..

[35] Vafadari R., Kraaijeveld R., Weimar W., Baan C.C. (2013). Tacrolimus Inhibits NF-ΚB Activation in Peripheral Human T Cells. PLoS ONE.

[36] Bendíčková K., Tidu F., De Zuani M., Kohoutková M.H., Andrejčinová I., Pompeiano A., Bělášková S., Forte G., Zelante T., Frič J. (2020). Calcineurin Inhibitors Reduce NFAT-Dependent Expression of Antifungal Pentraxin-3 by Human Monocytes. J. Leukoc. Biol..

[37] Du S., Hiramatsu N., Hayakawa K., Kasai A., Okamura M., Huang T., Yao J., Takeda M., Araki I., Sawada N. (2009). Suppression of NF-KappaB by Cyclosporin a and Tacrolimus (FK506) via Induction of the C/EBP Family: Implication for Unfolded Protein Response. J. Immunol..

[38] Lin H.Y.H., Chang K.T., Hung C.C., Kuo C.H., Hwang S.J., Chen H.C., Hung C.H., Lin S.F. (2014). Effects of the MTOR Inhibitor Rapamycin on Monocyte-Secreted Chemokines. BMC Immunol..

[39] Chaintreuil P., Kerreneur E., Bourgoin M., Savy C., Favreau C., Robert G., Jacquel A., Auberger P. (2023). The Generation, Activation, and Polarization of Monocyte-Derived Macrophages in Human Malignancies. Front. Immunol..

[40] Jiao B., An C., Du H., Tran M., Wang P., Zhou D., Wang Y. (2021). Stat6 Deficiency Attenuates Myeloid Fibroblast Activation and Macrophage Polarization in Experimental Folic Acid Nephropathy. Cells.

[41] Jiao B., An C., Tran M., Du H., Wang P., Zhou D., Wang Y. (2021). Pharmacological Inhibition of STAT6 Ameliorates Myeloid Fibroblast Activation and Alternative Macrophage Polarization in Renal Fibrosis. Front. Immunol..

[42] An C., Jiao B., Du H., Tran M., Song B., Wang P., Zhou D., Wang Y. (2023). Jumonji Domain-Containing Protein-3 (JMJD3) Promotes Myeloid Fibroblast Activation and Macrophage Polarization in Kidney Fibrosis. Br. J. Pharmacol..

[43] Han X., Li L., Yang J., King G., Xiao Z., Quarles L.D. (2016). Counter-Regulatory Paracrine Actions of FGF-23 and 1,25(OH)2D in Macrophages. FEBS Lett..

[44] Rajasekaran M., Sul O.J., Choi E.K., Kim J.E., Suh J.H., Choi H.S. (2019). MCP-1 Deficiency Enhances Browning of Adipose Tissue via Increased M2 Polarization. J. Endocrinol..

[45] Xu M., Wang X., Li Y., Geng X., Jia X., Zhang L., Yang H. (2021). Arachidonic Acid Metabolism Controls Macrophage Alternative Activation Through Regulating Oxidative Phosphorylation in PPARγ Dependent Manner. Front. Immunol..

[46] Simopoulos A.P. (2002). The Importance of the Ratio of Omega-6/Omega-3 Essential Fatty Acids. Biomed. Pharmacother..

[47] Wang T., Fu X., Chen Q., Patra J.K., Wang D., Wang Z., Gai Z. (2019). Arachidonic Acid Metabolism and Kidney Inflammation. Int. J. Mol. Sci..

[48] Hanna V.S., Hafez E.A.A. (2018). Synopsis of Arachidonic Acid Metabolism: A Review. J. Adv. Res..

[49] Martinelli N., Girelli D., Malerba G., Guarini P., Illig T., Trabetti E., Sandri M., Friso S., Pizzolo F., Schaeffer L. (2008). FADS Genotypes and Desaturase Activity Estimated by the Ratio of Arachidonic Acid to Linoleic Acid Are Associated with Inflammation and Coronary Artery Disease. Am. J. Clin. Nutr..

[50] Krüger B., Schröppel B., Ashkan R., Marder B., Zülke C., Murphy B., Krämer B.K., Fischereder M. (2002). A Monocyte Chemoattractant Protein-1 (MCP-1) Polymorphism and Outcome after Renal Transplantation. J. Am. Soc. Nephrol..

[51] Jang H.R., Kim M., Hong S., Lee K., Park M.Y., Yang K.E., Lee C.J., Jeon J., Lee K.W., Lee J.E. (2021). Early Postoperative Urinary MCP-1 as a Potential Biomarker Predicting Acute Rejection in Living Donor Kidney Transplantation: A Prospective Cohort Study. Sci. Rep..

